# Supplementary material for: A bacterial small RNA regulates the adaptation of Helicobacter pylori to the host environment
Source: Nat Commun. 2021 Apr 9;12:2085. doi: 10.1038/s41467-021-22317-7 (PMC8035401; doi:10.1038/s41467-021-22317-7)
Supplement: Supplementary file 1 — Supplementary Information [file 41467_2021_22317_MOESM1_ESM.pdf]

Supplementary Information

A bacterial small RNA regulates the adaptation of *Helicobacter pylori* to the host environment

Ryo Kinoshita-Daitoku, Kotaro Kiga, Masatoshi Miyakoshi, Ryota Otsubo, Yoshitoshi Ogura, Takahito Sanada, Zhu Bo, Tuan Vo Phuoc, Tokuju Okano, Tamako Iida, Rui Yokomori, Eisuke Kuroda, Sayaka Hirukawa, Mototsugu Tanaka, Arpana Sood, Phawinee Subsomwong, Hiroshi Ashida, Tran Thanh Binh, Lam Tung Nguyen, Khien Vu Van, Dang Quy Dung Ho, Kenta Nakai, Toshihiko Suzuki, Yoshio Yamaoka, Tetsuya Hayashi, and Hitomi Mimuro

Correspondence and requests for materials should be addressed to Hitomi Mimuro (mimuro@biken.osaka-u.ac.jp)

This PDF file includes:

Supplementary Figures 1 to 7

Other Supplementary Information files in this study include the following:

Supplementary Data 1 to 8

a

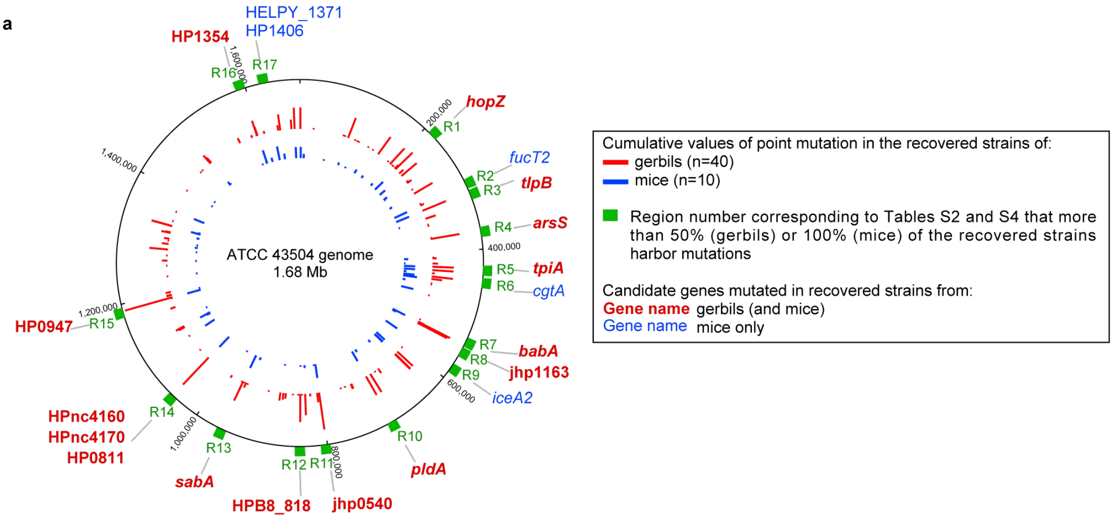

b

Region No.	Gene symbol	Gene description	Mongolian gerbils (8 wks post infection)		C57BL/6 mice (8 wks post infection)	
			Fold change of mRNA expression level [mut(+)/input]	Mutation rate (%, /40 strains)	Fold change of mRNA expression level [mut(+)/input]	Mutation rate (%, /10 strains)
R1	*hopZ*	outer membrane protein	0.373	52.5	N/A	N/A
R2	*fucT2*	alpha-1,2-fucosyltransferase	N/A	N/A	0.758	100.0
R3	*tlpB*	methyl-accepting chemotaxis protein	1.239	50.0	0.913	100.0
R4	*arsS*	histidine kinase sensor protein	3.572	62.5	N/A	N/A
R5	*tpiA*	triosephosphate isomerase	1.286	80.0	0.825	100.0
R6	*cgtA*	beta-1,4-N-acetylgalactosamyltransferase	N/A	N/A	1.453	100.0
R7	*babA*	outer membrane protein babA	1.263	80.0	1.243	100.0
R8	jhp1163	hypothetical protein	1.374	80.0	N/A	N/A
R9	iceA2	Ulcer-associated gene restriction endonuclease	N/A	N/A	1.104	100.0
R10	*pldA*	phospholipase A1	2.420	60.0	N/A	N/A
R11	jhp0540	hypothetical protein	1.946	70.0	1.497	100.0
R12	HPB8_818	family 25 glycosyl transferase	5.346	52.5	N/A	N/A
R13	*sabA*	outer membrane protein sabA	5.215	62.5	1.512	100.0
R14	HPnc4160	mRNA/antisense RNA family IsoB	0.155	72.5	0.297	100.0
R14	HPnc4170	mRNA/antisense RNA family aapB	0.794	72.5	1.043	100.0
R14	HP0811	hypothetical protein	1.698	72.5	1.075	100.0
R15	HP0947	hypothetical protein	1.300	92.5	0.511	100.0
R16	HP1354	adenine-specific DNA methyltransferase	1.512	57.5	1.138	100.0
R17	HELPY_1371	Type III restriction enzyme R protein	N/A	N/A	1.018	100.0
R17	HP1406	biotin synthase	N/A	N/A	1.200	100.0

c

Strainns from C57BL/6 mice infected with ATCC 43504 WT

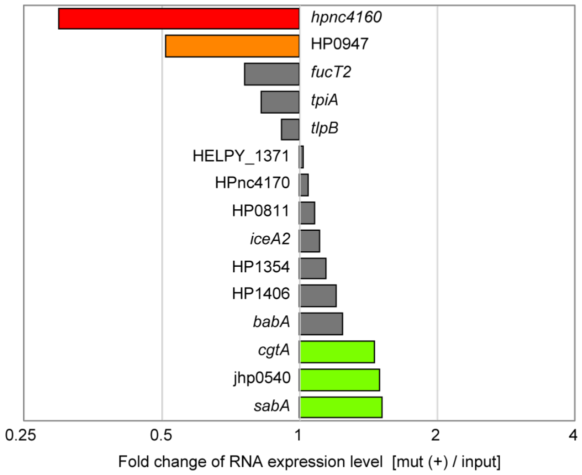

Supplementary Fig. 1 Candidate RNA expression of strains recovered from *H. pylori* ATCC 43504-infected rodent stomachs. a, Circular genomic map of ATCC 43504 strain recovered from stomachs of gerbils and mice. **b**, Mutation rates and expression levels of candidate RNAs (mRNA or non-coding RNA) of strains recovered from the stomachs of gerbils or mice 8 weeks post-infection. The locus tags (red) indicate candidates common in both strains originating from gerbils and mice. N/A, not applicable. **c**, Candidate RNA (mRNA or non-coding RNA) expression of isolates recovered from *H. pylori* ATCC 43504-infected C57BL/6 mice stomachs. RNA expression of the genes or nearby genes of genome regions (Supplementary Fig. 1a and Supplementary Data 4), which were mutated in 100% of the recovered strains were assessed. Source data are provided as a Source Data file.

a

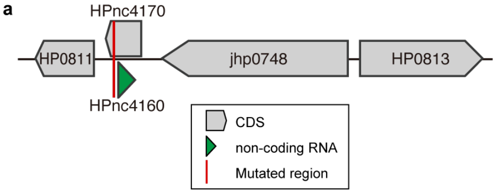

b

```
                           -40       -30       -20            -10       1         10        20             30        40
     H. pylori ATCC 43504  aaataacatcgtttttttttttttt-----ggtataatgctcggcgaaggtaagagcgaaaggcgtattata----ttccttccttctt
          H. pylori PMSS1  aaataacatc-tttttttttttt-------ggtataatgc-cgcctaaggtaagagcgaaaggcgtattata----ttccttccttctt
            H. pylori SS1  aaataacatc-ttttttttttttt------ggtataatgc-cgcctaaggtaagagcgaaaggcgtattata----ttccttccttctt
          H. pylori 26695  aaataacatcgtttttttttttttt-----ggtataatgctcggcgaaggtaagagcgaaaggcgtattata----ttccttctttctt
            H. pylori J99  aaataacatcgtttttttttttttt-----ggtataatgctcggcgaaggtaagagcgaaaggcgtattata----ttccttccttctt
          H. pylori HPAG1  aaataacatcgttttttttttttttttt--ggtataatgctcggcgaaggtaagagcgaaaggcgtattata----ttccttccttctt
            H. pylori G27  aaataacatc-tttttttttttttttt---ggtataatgc-cgcttaaggtaagagcgaaaggcgtattata----ttccttccttctt
         H. pylori Shi470  aaataacatcgtttttttttttttt-----ggtataatgctcggtgaaggtaagagcaaaggcgtattatattccttccttccttctt
H. acinonychis Sheeba      aaataaca---tttttttttttagtataatagtataatgt-tgttg--ggtaagggcaaagggcg-aaaata----ttccttccttctt

                           41        50         60        70                80        90        100
     H. pylori ATCC43504   tactataac-ttagca-ttttaatcaacttttt-------cattaaaatgtcctgacgctcttacctt-aa
          H. pylori PMSS1  tactataac-ttagca-ttttaatcaacttttt-------cattaaaatgtcctgacgctcttacctttc
          H. pylori   SS1  tactataac-ttagca-ttttaatcaacttttt-------cattaaaatgtcctgacgctcttacctttc
          H. pylori 26695  tactataac-ttagca-ttttaatcaacttttt-------cattaaaatgtcctgacgctcttacctt-aa
          H. pylori   J99  tactataac-ttagca-ttttaatcaacttttt-------cattaaaatgtcctgacgctcttaccttaaa
          H. pylori HPAG1  tactataac-ttagca-ttttaatcaacttttt-------cattaaaatgtcctgacgctcttacctt-aa
          H. pylori   G27  tactataac-ttagca-ttttaatcaacttttt-------cattaaaatgtcctgacgctcttacctt---
         H. pylori Shi470  tactataac-ttagca-ttttaataaacttttt-------cattaaaatgtcctgacgctcttaccttcaa
 H. acinonychis Sheeba     tactataacattagcattttagtaaactttttctttttacattaaaatgtcctaatgctcttacctt--a
```

c ATCC 43504 WT

```
-40       -30       -20       -10       1         10        20        30        40        50
aaataacatcgttttttttttttttggtataatgctcggcgaaggtaagagcgaaaggcgtattatattccttccttctttactataactta...
```

T-repeat elongation pattern in the recovered strains from mice

No. of strains/total	
1/10 (10%)	GTTTTTTTTTTTTTTT
5/10 (50%)	GTTTTTTTTTTTTTTTT
4/40 (40%)	GTTTTTTTTTTTTTTTTT

Green : HPnc4160 transcribed sequence
Red frame : T-repeat region
Red Ts : inserted nucleotides

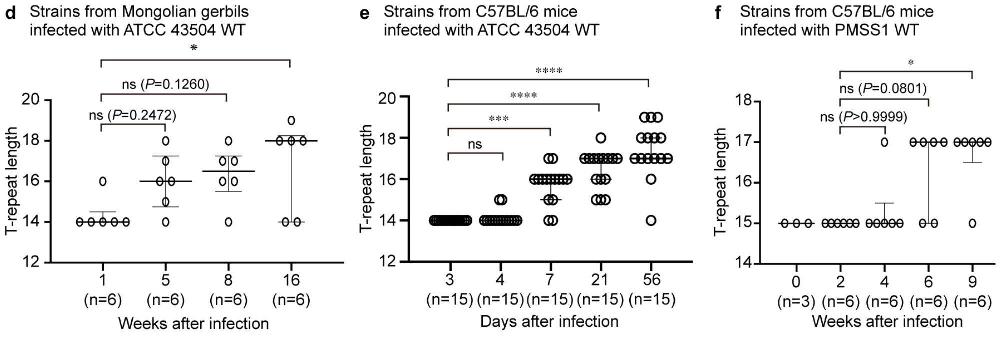

g

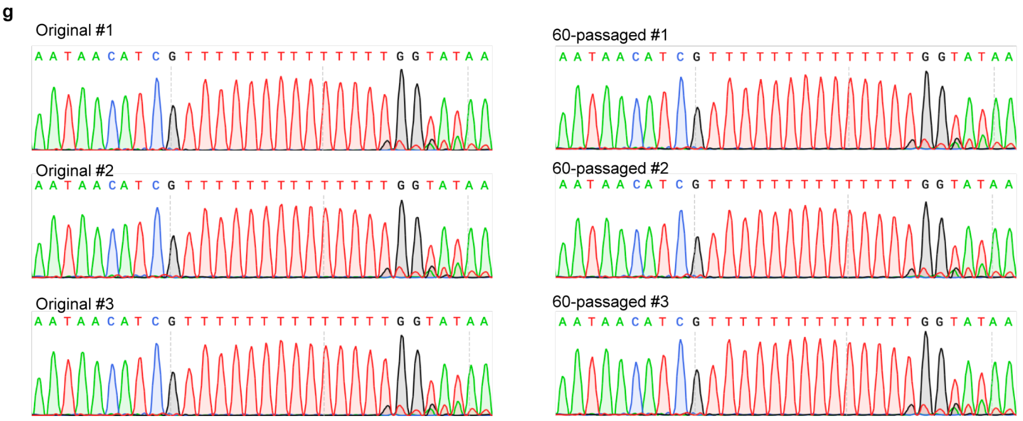

Supplementary Fig. 2 Length of the T-repeat region upstream of the HPnc4160 coding region. a, Schematic structures of genes around HPnc4160. **b**, Schematic of the DNA sequence around the HPnc4160 and T-repeat sequences of analyzed strains. Red ts = T-repeat sequences. **c**, Schematic of the DNA sequence around the HPnc4160 and T-repeat sequence of mice-recovered strains 8 wks after infection (n = 10). Green = HPnc4160 transcribed sequence, red frame = T-repeat region upstream of HPnc4160, and red- Ts = inserted nucleotides compared with wild-type. **d-f**, Time-dependent changes in the length of the T-repeat region upstream of the HPnc4160 coding region in gerbils or mice-recovered strains. Strains from Mongolian gerbils infected with ATCC 43504 (**d**). Strains from C57BL/6 mice infected with ATCC 43504 (**e**). Strains from C57BL/6 mice infected with PMSS1 (**f**). Data are presented as the median with interquartile range. *P* values were obtained from non-parametric Dunn's multiple comparison test (two-sided). *$P = 0.0373$ (**d**); ***$P = 0.0007$, ****$P < 0.0001$ (**e**); *$P = 0.0130$ (**f**); ns: not significant. **g**, Effect of *in vitro* cultivation on the length of the T-repeat region upstream of the HPnc4160 coding region. Raw data from the DNA sequence analysis of *H. pylori* genomes prepared from original culture (Original #1 - #3) and from those passaged *in vitro* 60 times (60-passaged #1 - #3). Source data are provided as a Source Data file.

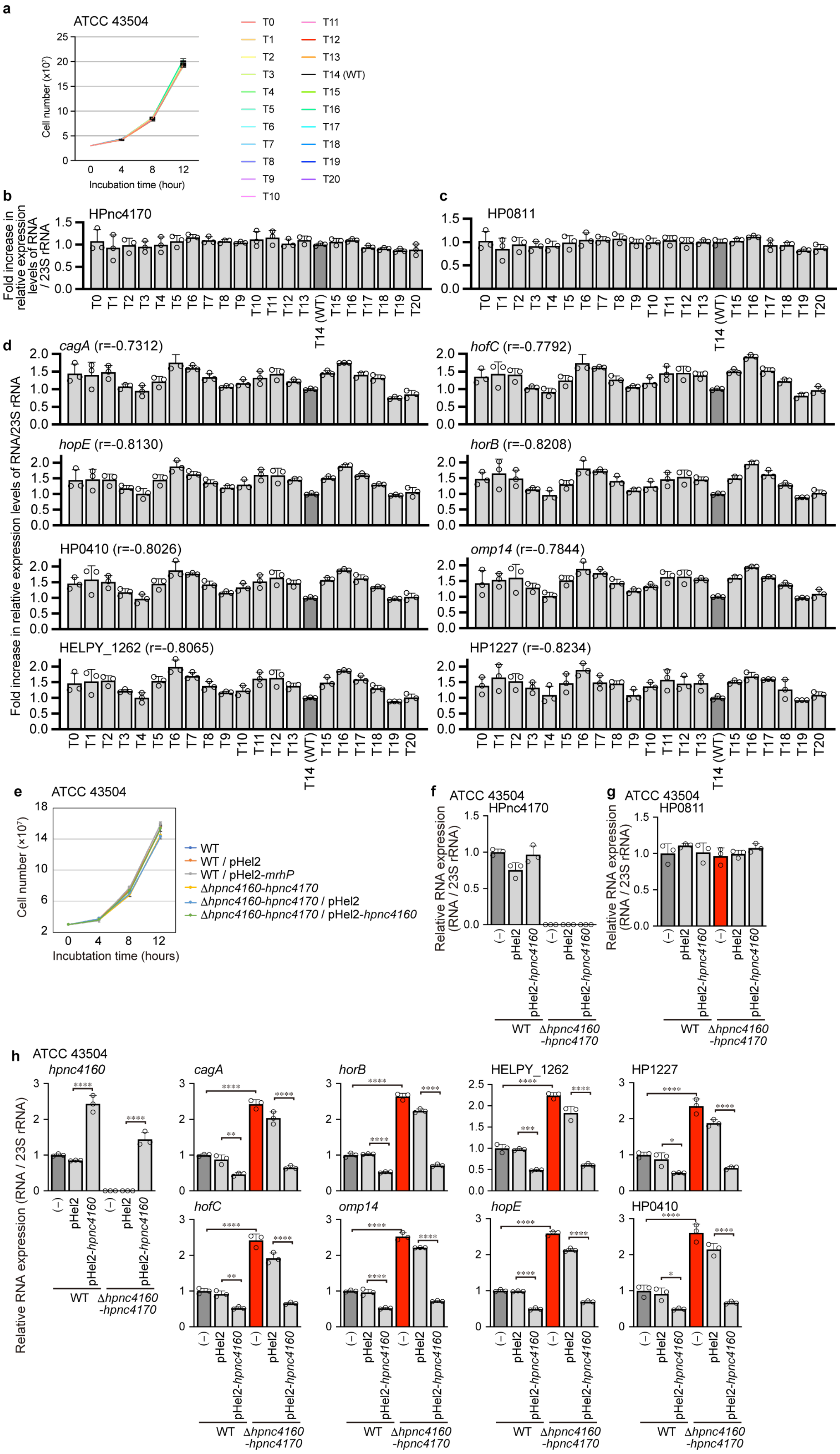

a
ATCC 43504
Cell number (x10⁷)
Incubation time (hour)
T0
T1
T2
T3
T4
T5
T6
T7
T8
T9
T10
T11
T12
T13
T14 (WT)
T15
T16
T17
T18
T19
T20
b
HPnc4170
Fold increase in relative expression levels of RNA / 23S rRNA
c
HP0811
d
cagA (r=-0.7312)
hofC (r=-0.7792)
hopE (r=-0.8130)
horB (r=-0.8208)
HP0410 (r=-0.8026)
omp14 (r=-0.7844)
HELPY_1262 (r=-0.8065)
HP1227 (r=-0.8234)
Fold increase in relative expression levels of RNA/23S rRNA
e
ATCC 43504
Cell number (×10⁷)
Incubtation time (hours)
WT
WT / pHel2
WT / pHel2-mrhP
Δhpnc4160-hpnc4170
Δhpnc4160-hpnc4170 / pHel2
Δhpnc4160-hpnc4170 / pHel2-hpnc4160
f
ATCC 43504
HPnc4170
Relative RNA expression (RNA / 23S rRNA)
(-)
pHel2
pHel2-hpnc4160
WT
Δhpnc4160 -hpnc4170
g
ATCC 43504
HP0811
h
ATCC 43504
hpnc4160
cagA
horB
HELPY_1262
HP1227
hofC
omp14
hopE
HP0410
Relative RNA expression (RNA / 23S rRNA)

Supplementary Fig. 3 Validation of target mRNA of HPnc4160, and the effect of HPnc4160, upstream T-repeat length on mRNA expression. a, Growth curves of *H. pylori* ATCC 43504 mutants with respect to the number of T-repeats upstream of the HPnc4160 coding region. **b**, **c**, Relative expression of *hpnc4170* (**b**) and HP0811 (**c**) in the *H. pylori* strains genetically modified with respect to T-repeat length. Data are presented as means ± s.d. (n = 3). **d**, Total RNA from the indicated *H. pylori* ATCC 43504 strains was extracted, reverse transcribed and analyzed by qPCR to assess the indicated genes. Data are presented as the means ± s.d. of three separate experiments. Spearman correlation coefficients (r) were used to evaluate the relationship between the relative RNA expression of HPnc4160 (Fig. 1e) and that of each target. **e**, Growth curves of *H. pylori* ATCC 43504 mutants. **f–h**, Relative RNA expression of HPnc4170 (**f**), HP0811 (**g**), and target candidates for HPnc4160 (**h**). Data are presented as the means ± s.d. of three experiments. **P = 0.0043 (cag*A*), ***P = 0.0001 (HELPY_1262), *P = 0.0226 (HP1227), **P = 0.0072 (*hofC*), *P = 0.0483 (HP0410); ****P < 0.0001, by Tukey's multiple comparison test (two-sided). Data are representative of two independent experiments (**a**-**h**). Source data are provided as a Source Data file.

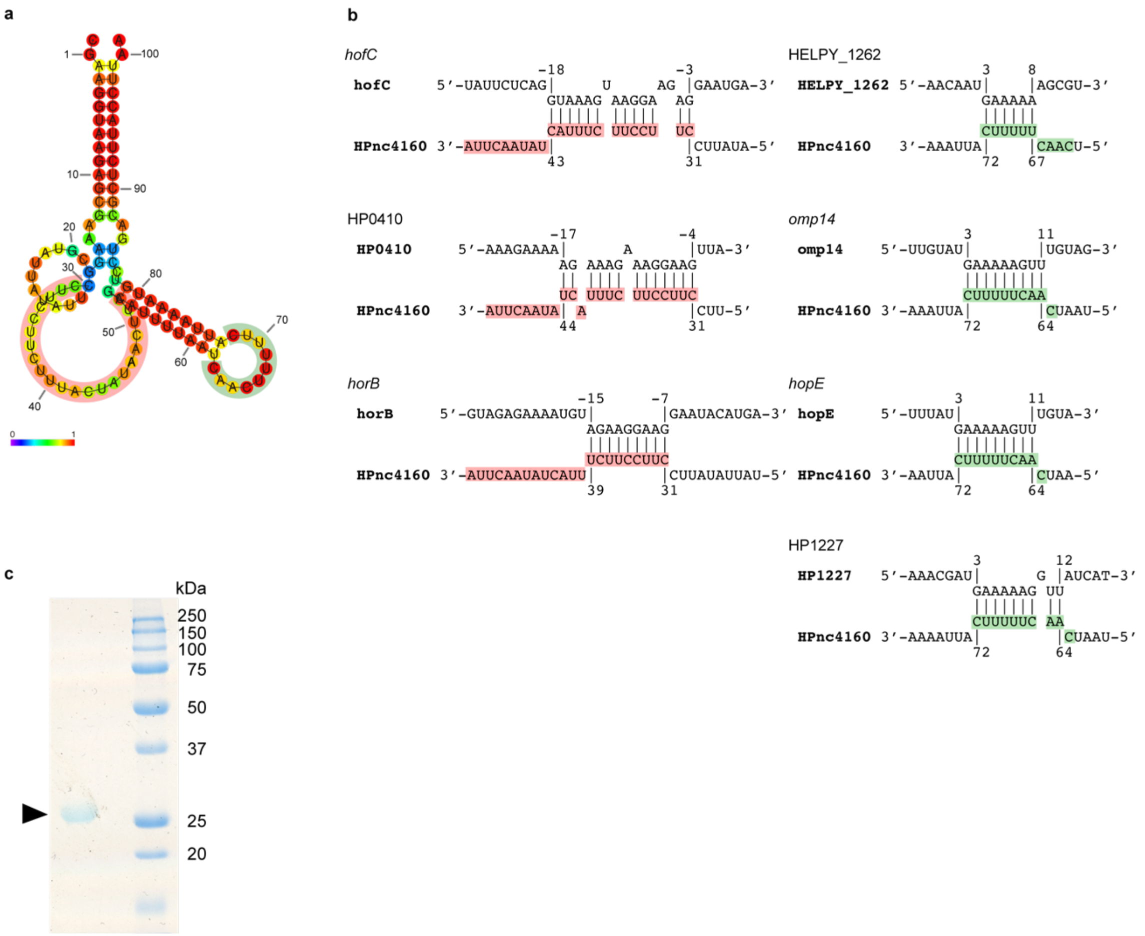

Supplementary Fig. 4 Predicted HPnc4160 binding sites. a, Predicted HPnc4160 binding sites. **a**, Secondary structure of HPnc4160 RNA as predicted by CentroidFold. The bases in the predicted structure are colored according to base-pairing probabilities. Circles in pink and light green indicate loop structures with probable binding to target RNA sequences. **b**, Schematic of predicted HPnc4160 binding sites in the corresponding 5′UTR sequence of target genes. Upper sequences indicate target mRNA sequences with base numbers, whereas lower sequences indicate the HPnc4160 sequence. Colored sequences correspond to the loop structures indicated in (**a**). **c**, Purified RNase III was separated by SDS-PAGE and stained with CBB. Data are representative of two independent experiments (**c**). Source data are provided as a Source Data file.

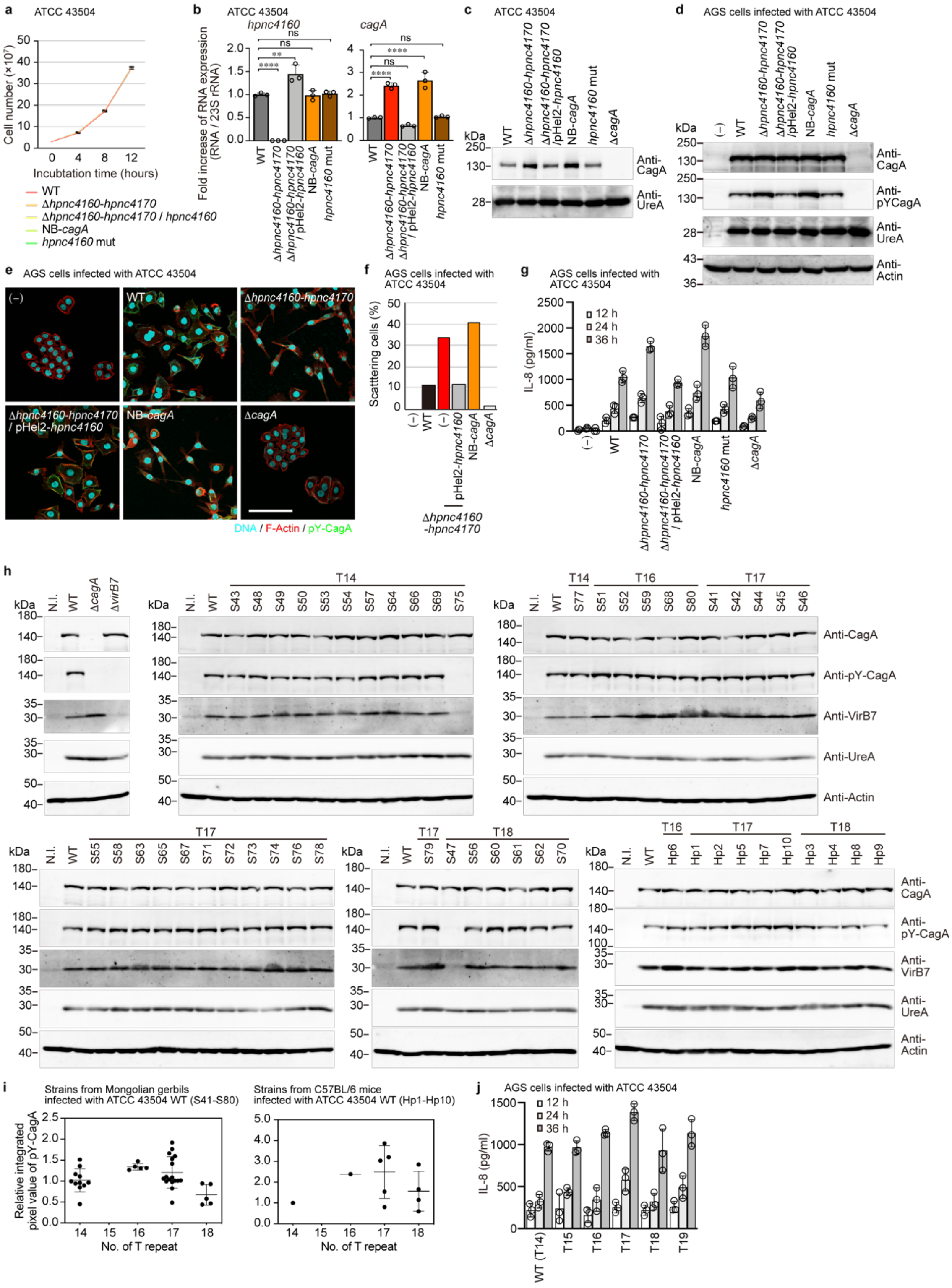

Supplementary Fig. 5 Effect of NB-*cagA* on host-cell-translocated CagA activity. a, Growth curves of *H*. *pylori* ATCC 43504 NB-*cagA* mutant compared with wild-type. **b**, Relative RNA expression of HPnc4160 and *cagA* in each mutant strain. Data are

presented as the means ± s.d. of three experiments. ***P* = 0.0013, *****P* < 0.0001 by non-parametric Dunnett's multiple comparison test (two-sided). ns: not significant. **c**, CagA levels in each mutant strain, UreA served as the bacterial loading control. **d**, Phosphorylated CagA protein levels in cell lysates of AGS cells infected with *H. pylori* ATCC 43504. Whole-cell lysates of AGS cells infected with *H. pylori* strains for 6 h were subjected to western blot against anti-CagA, anti-pY CagA, anti-UreA, and anti-actin antibodies. Actin served as the cellular loading control. **e**–**f**, Scattering phenotypes of *H. pylori* ATCC 43504-infected AGS gastric epithelial cells. **e**, DNA (blue), F-actin (red), and anti-phosphorylated CagA antibody (pY-CagA, green). Scale bar, 50 μm. **f**, Quantification of scattering activity of AGS cells induced by *H. pylori* infection. **g**,**j**, Supernatants from AGS cells infected with *H. pylori* strains for the indicated time were subjected to ELISA for IL-8. Data are presented as the means ± s.d. of three independent experiments. **h**–**i**, Phosphorylated CagA protein and VirB7 levels in cell lysates of AGS cells infected with *H. pylori* isolates from rodents. Whole-cell lysates of AGS cells infected with *H. pylori* strains (S41–S80, strains from Mongolian gerbils infected with ATCC 43504 WT; Hp1–Hp10, strains from C57BL/6 mice infected with ATCC 43504 WT; Supplementary Data 1 and 3) for 6 h were subjected to western blot against anti-CagA, anti-pY CagA, anti-UreA, anti-actin, and anti-VirB7 antibodies. The band intensities (excluding S47 and S75 lacking VirB7) were measured and calculated by ImageJ software (**i**). Data are representative of two independent experiments (**a**-**j**). Source data are provided as a Source Data file.

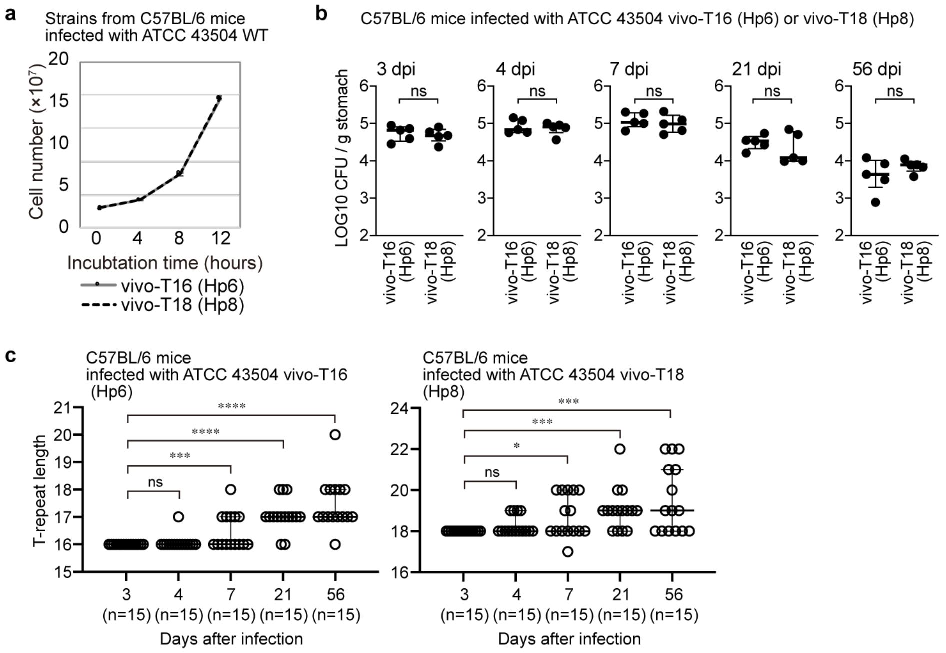

Supplementary Fig. 6 Time-dependent changes in bacterial-host adaptation and the T-repeat length of isolates from mice. a, Growth curves of mouse-derived strains Hp6 and Hp8 recovered from mouse at 8 wks post infection (Hp6, possessing T16 repeat; Hp8, possessing T18 repeat; Supplementary Data 4 and 5). Data are representative of two independent experiments. **b–c**, Reinfection of isolates from mouse stomach (Hp6, possessing T16 repeat; Hp8, possessing T18 repeat). Bacterial number in mouse stomach (**b**) and the length of the T-repeat region upstream of the HPnc4160 coding region (**c**) in isolates from mouse stomach infected with Hp6 or Hp8 were measured. Statistical significance determined by two-tailed Mann-Whitney test (**b**), or uncollected two-tailed Dunn's multiple comparison test (**c**). ***$P = 0.00479$, ****$P < 0.0001$ (Hp6); *$P = 0.0189$, ***$P = 0.0004$ (middle and upper), (Hp8); ns, not significant. Source data are provided as a Source Data file.

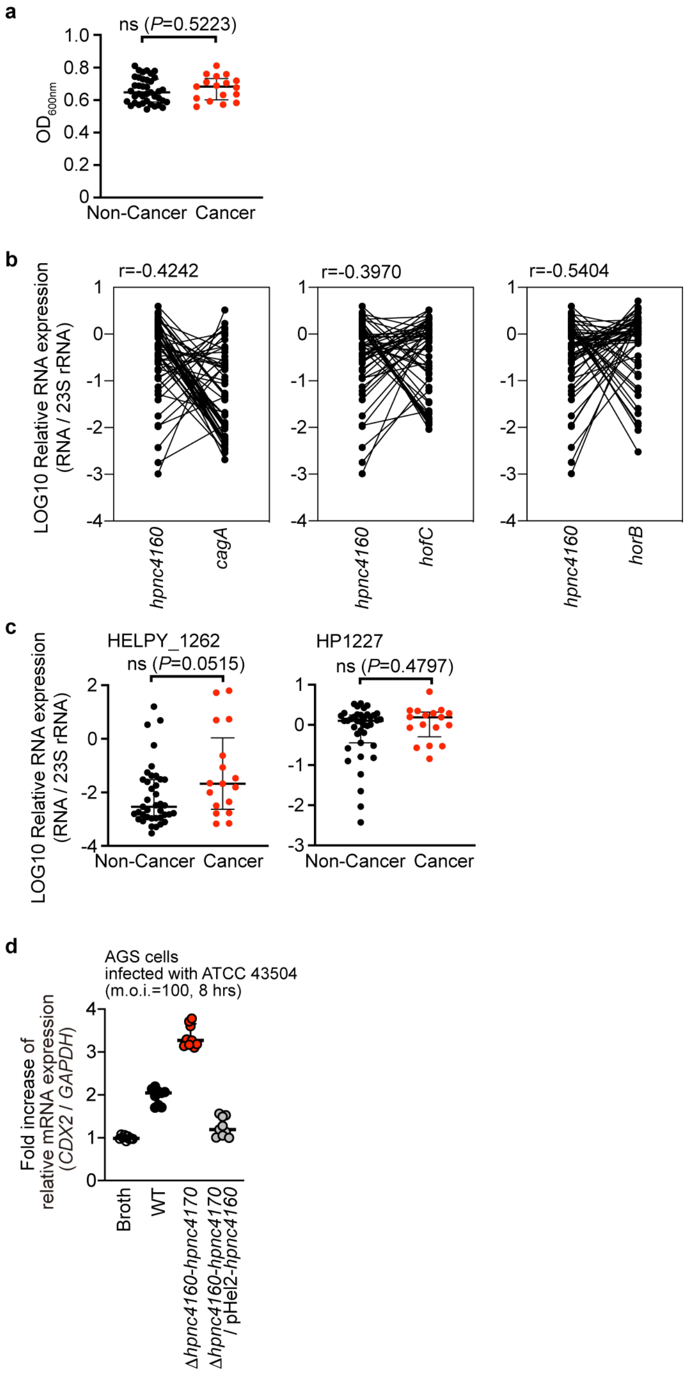

Supplementary Fig. 7 Characterization of clinical isolates and effect of HPnc4160 on expression of *CDX2*, a marker for the intestinal-type gastric epithelial neoplasia. **a**, Growth rates of clinical isolates of non-cancer patients (non-cancer, n = 39) and patients with cancer (cancer, n = 17), as used in Fig. 4d. Data are presented as the medians with interquartile range. *P* values represent the results of a two-tailed Mann-Whitney test. ns: not significant. **b**, Relative RNA expression of candidate HPnc4160 targets. Spearman

correlation coefficients (r) were used to evaluate the relationships between the relative RNA expression of HPnc4160 and that of each target. **c**, Comparison of mRNA (HELPY_1262 and HP1227) expression in clinical isolates of non-cancer patients (non-cancer, n = 39) and patients with cancer (cancer, n = 17). mRNA expression was normalized to that of 23S rRNA. **d**, Expression levels of *CDX2* in AGS cells infected with indicated *H. pylori* at 8 hours post infection at multiplicity of infection (m.o.i.) of 100. Data are presented as the medians with interquartile range. *P* values represent the results of a two-tailed Mann-Whitney test. Source data are provided as a Source Data file.
