# Supplementary material for: A bacterial small RNA regulates the adaptation of Helicobacter pylori to the host environment
Source: Nat Commun. 2021 Apr 9;12:2085. doi: 10.1038/s41467-021-22317-7 (PMC8035401; doi:10.1038/s41467-021-22317-7)
Supplement: Supplementary file 3 — Description of Additional Supplementary Files [file 41467_2021_22317_MOESM3_ESM.pdf]

Description of Additional Supplementary Files

File Name: Supplementary Data 1
Description: Summary of mutations in the isolates recovered from *H. pylori*-infected Mongolian gerbils. The number of mutations in the isolates of 40 strains recovered from *H. pylori*-infected Mongolian gerbils' stomachs 8 weeks post infection are listed.

File Name: Supplementary Data 2
Description: Mutated sequence list of the 40 strains recovered from *H. pylori*-infected Mongolian gerbils' stomach.

File Name: Supplementary Data 3
Description: Summary of mutations in the isolates recovered from *H. pylori*-infected C57BL/6 mice. The number of mutations in the isolates of 10 strains recovered from *H. pylori*-infected mice stomachs 8 weeks post-infection are listed.

File Name: Supplementary Data 4
Description: Mutated sequence list of the 10 strains recovered from *H. pylori*-infected C57BL/6 mice stomachs.

File Name: Supplementary Data 5
Description: Comparative analysis of RNA expression levels between *H. pylori* ATCC 43504 Δhpnc4160-hpnc4170 mutant and wild-type strains by RNA-seq. Comparative analysis of RNA expression levels between *H. pylori* ATCC 43504 Δ*hpnc4160-hpnc4170* mutant and wild-type strains by RNA-seq. Normalized expression level and fold change of the strains are listed. Genes with $P < 0.001$ by Empirical Analysis of Digital Gene Expression in R (edgeR) test are listed (17 factors). The eight genes selected by RNA-seq and iTRAQ analysis (Fig. 2c) are highlighted in red.

File Name: Supplementary Data 6
Description: Comparative analysis of protein expression levels between *H. pylori* ATCC 43504 Δ*hpnc4160-hpnc4170* mutant and wild-type strains by iTRAQ.

File Name: Supplementary Data 7
Description: Information of the *H. pylori* clinical isolates used in Fig. 6 and Supplementary Fig. 7. Comparative analysis of protein expression levels between *H. pylori* ATCC 43504 Δ*hpnc4160-hpnc4170* mutant and wild-type strains by iTRAQ. Proteins showing relative protein abundance with $P < 0.01$ are listed (21 factors). The eight proteins selected by RNA-seq and iTRAQ analysis (Fig. 2c) are highlighted in red.

File Name: Supplementary Data 8
Description: Primers used in this study
