# Supplementary material for: A bacterial small RNA regulates the adaptation of Helicobacter pylori to the host environment
Source: Nat Commun. 2021 Apr 9;12:2085. doi: 10.1038/s41467-021-22317-7 (PMC8035401; doi:10.1038/s41467-021-22317-7)
Supplement: Supplementary file 12 — Reporting Summary [file 41467_2021_22317_MOESM12_ESM.pdf]

nature research

Corresponding author(s): Hitomi Mimuro

Last updated by author(s): Mar 4, 2021

Reporting Summary

Nature Research wishes to improve the reproducibility of the work that we publish. This form provides structure for consistency and transparency in reporting. For further information on Nature Research policies, see our Editorial Policies and the Editorial Policy Checklist.

Statistics

For all statistical analyses, confirm that the following items are present in the figure legend, table legend, main text, or Methods section.

n/a	Confirmed	
☐	☒	The exact sample size (n) for each experimental group/condition, given as a discrete number and unit of measurement
☒	☐	A statement on whether measurements were taken from distinct samples or whether the same sample was measured repeatedly
☐	☒	The statistical test(s) used AND whether they are one- or two-sided<br>*Only common tests should be described solely by name; describe more complex techniques in the Methods section.*
☒	☐	A description of all covariates tested
☐	☒	A description of any assumptions or corrections, such as tests of normality and adjustment for multiple comparisons
☐	☒	A full description of the statistical parameters including central tendency (e.g. means) or other basic estimates (e.g. regression coefficient) AND variation (e.g. standard deviation) or associated estimates of uncertainty (e.g. confidence intervals)
☐	☒	For null hypothesis testing, the test statistic (e.g. F, t, r) with confidence intervals, effect sizes, degrees of freedom and P value noted<br>*Give P values as exact values whenever suitable.*
☒	☐	For Bayesian analysis, information on the choice of priors and Markov chain Monte Carlo settings
☒	☐	For hierarchical and complex designs, identification of the appropriate level for tests and full reporting of outcomes
☐	☒	Estimates of effect sizes (e.g. Cohen's d, Pearson's r), indicating how they were calculated

Our web collection on statistics for biologists contains articles on many of the points above.

Software and code

Policy information about availability of computer code

Data collection	Illumina MiSeq, HiSeq X, Illumina Hiseq 2500 Genome Analyzer, ZEN (ver. 2.3)
Data analysis	Graphpad Prism (ver. 8), Platanus assembler (ver. 1.2.1), ProteinPilot software (ver. 5.0), Tophat (ver. 2.1.0), ImageJ (ver. 1.52a)

For manuscripts utilizing custom algorithms or software that are central to the research but not yet described in published literature, software must be made available to editors and reviewers. We strongly encourage code deposition in a community repository (e.g. GitHub). See the Nature Research guidelines for submitting code & software for further information.

Data

Policy information about availability of data

All manuscripts must include a data availability statement. This statement should provide the following information, where applicable:

- Accession codes, unique identifiers, or web links for publicly available datasets
- A list of figures that have associated raw data
- A description of any restrictions on data availability

The raw read sequences and assembled scaffold sequences of H. pylori strains recovered from rodents have been deposited to the DDBJ/EMBL/Genbank under the Bioproject accession numbers; SAMD00178897- SAMD00178935, SAMD00179460, SAMD00178937, and SAMD00204457- SAMD00204466 (https://ddbj.nig.ac.jp/BSSearch/). Sequence data of clinical isolates used in this study have been available in the DDBJ/EMBL/Genbank with the accession codes listed in Supplementary Data 7. The authors declare that all other data supporting the findings of this study are available with the paper and its supplementary Data files.

Field-specific reporting

Please select the one below that is the best fit for your research. If you are not sure, read the appropriate sections before making your selection.

- [x] Life sciences
- [ ] Behavioural & social sciences
- [ ] Ecological, evolutionary & environmental sciences

For a reference copy of the document with all sections, see nature.com/documents/nr-reporting-summary-flat.pdf

Life sciences study design

All studies must disclose on these points even when the disclosure is negative.

Sample size	No statistical methods were used to predetermine sample size. Sample size was chosen by following the literature in the field, our preliminary experiments and experience.
Data exclusions	Before aligned RNA-seq reads to the corresponding ATCC 43504 genome using Tophat 2.1.0, we removed the low-quality reads and adaptors from the paired-end 150-bp reads generated by high-throughput sequencing with an Illumina Hiseq 2500 Genome Analyzer. Data were processed by Grubbs' test for outliers (no applicable data, Figure 1d).
Replication	All experiments (excluding iTRAQ data) were performed at least two independent biological replicates. All the attempts at replication were successful.
Randomization	Randomization is not applicable since there are no group allocation in this study.
Blinding	Blinding is not applicable since there are no group allocation in this study.

Reporting for specific materials, systems and methods

We require information from authors about some types of materials, experimental systems and methods used in many studies. Here, indicate whether each material, system or method listed is relevant to your study. If you are not sure if a list item applies to your research, read the appropriate section before selecting a response.

Materials & experimental systems

n/a	Involved in the study	
[ ]	[x]	Antibodies
[ ]	[x]	Eukaryotic cell lines
[x]	[ ]	Palaeontology and archaeology
[ ]	[x]	Animals and other organisms
[x]	[ ]	Human research participants
[x]	[ ]	Clinical data
[x]	[ ]	Dual use research of concern

Methods

n/a	Involved in the study	
[x]	[ ]	ChIP-seq
[x]	[ ]	Flow cytometry
[x]	[ ]	MRI-based neuroimaging

Antibodies

Antibodies used	Anti-Tyr(P)-CagA, anti-UreA, and anti-VirB7 polyclonal antibodies (Mimuro, H. et al. Helicobacter pylori Dampens Gut Epithelial Self-Renewal by Inhibiting Apoptosis, a Bacterial Strategy to Enhance Colonization of the Stomach. Cell Host Microbe 2, 250–263 (2007).), anti-CagA polyclonal antibody (AUSTRAL Biologicals; Product Number, HPP-5003-9), anti-actin monoclonal antibody (MERCK; Product Number, MAB1501), horseradish peroxidase (HRP)-labeled anti-rabbit IgG (SIGMA; Product Number, A 0545) and HRP-labeled anti-mouse IgG (SIGMA; Product Number, A 4416), fluorescein isothiocyanate-labeled anti-rabbit IgG (SIGMA; Product Number, F9887), Human IL-8 ELISA Kit (ThermoFisher SCIENTIFIC; Product Number, 88-8086-88).
Validation	All antibodies used in our study have been validated. Detailed information could be found on the manufactures' websites as listed below. Anti-actin monoclonal antibody has been validated by estimated molecular weight and immunohistochemical staining pattern (https://www.merckmillipore.com/JP/ja/product/Anti-Actin-Antibody-clone-C4,MM_NF-MAB1501).<br>Horseradish peroxidase (HRP)-labeled anti-rabbit IgG (https://www.sigmaaldrich.com/catalog/product/sigma/a0545?lang=ja&region=JP), HRP-labeled anti-mouse IgG (https://www.sigmaaldrich.com/catalog/product/sigma/a4416?lang=ja&region=JP), and fluorescein isothiocyanate-labeled anti-rabbit IgG (https://www.sigmaaldrich.com/catalog/product/sigma/f9887?lang=ja&region=JP) has been validated for Western blot.<br>Human IL-8 ELISA kit (https://www.thermofisher.com/elisa/product/IL-8-Human-Uncoated-ELISA-Kit/88-8086-88) has been validated by the standard curve of human recombinant IL-8.<br>Antibodies including CagA, pY-CagA, VirB7, and UreA have been validated by our experiments in this manuscript using WT, ΔcagA, ΔvirB7 strains of H. pylori and AGS cells without H. pylori infection.

Eukaryotic cell lines

Policy information about cell lines

Cell line source(s)	AGS cells were purchased from the American Type Culture Collection.
Authentication	The AGS cells have been authenticated by ATCC through STR profiling.
Mycoplasma contamination	The AGS cells we used were negative for mycoplasma contamination.
Commonly misidentified lines (See ICLAC register)	No commonly misidentified cell line was used.

Animals and other organisms

Policy information about studies involving animals; ARRIVE guidelines recommended for reporting animal research

Laboratory animals	6-week-old male MON/Jms/GbsSlc Mongolian gerbils and 6-week-old male C57BL/6 mice (SLC Japan Inc., Tokyo, Japan) were used. All the animals were housed under controlled conditions with a 12 h light/dark cycle, 20-22˚C and 45 ± 5% humidity.
Wild animals	None.
Field-collected samples	None.
Ethics oversight	Animal experiments were conducted in accordance with the University of Tokyo or Osaka University guidelines for the care and use of laboratory animals and were approved by the Ethics Committee for Animal Experiments at the University of Tokyo or Osaka University.

Note that full information on the approval of the study protocol must also be provided in the manuscript.
